# Impact of health awareness on myocardial infarction

**DOI:** 10.1186/s42506-025-00186-y

**Published:** 2025-03-27

**Authors:** Ahmed Magdy, Seham Elmarayed, Bassem Zarif, Mohamed Sabry, Ahmed Alsawah, Mohamed E. Hasan, Khaled M. Ismaeil, Mohamed Salama

**Affiliations:** 1 Global Health and Human Ecology Dept, The American University in Cairo, Cairo, Egypt; 2 Cardiology Dept, National Heart Institute, Giza, Egypt

**Keywords:** STEMI, Health awareness, Education, Time of presentation to the ER

## Abstract

**Background:**

Health awareness plays a major role in determining the outcomes of serious medical conditions especially when response time is crucial. STEMI (ST-segment elevation myocardial infarction) patients are prone to serious compilations if they do not receive the appropriate treatment on time. Many factors affect the health awareness of the community, including educational level, previous exposure to similar situations, and exposure to health awareness materials. Those who do not know the symptoms of myocardial infarction will present late to the hospital and are exposed to a higher risk of complications. This study aims to assess the relationship between the health awareness of STEMI patients and the time of presentation to the emergency room (ER).

**Methods:**

A cohort observational study was conducted at the National Heart Institute in Egypt gathering data on 263 STEMI patients presenting for primary percutaneous intervention. All the demographic and clinical necessary data was collected by the researchers in the emergency room, catheterization lab, and during the hospital admission of the patient. This study is a part of our larger cohort study on the relationship between education/health awareness of patients and outcomes of myocardial infarction.

**Results:**

Data from 166 eligible patients were analyzed showing a significant correlation between health awareness and time of presentation to the ER in STEMI patients (*p* < 0.05). Additionally, there was a significant correlation between educational level and time of presentation to the ER (*p* < 0.05). The mean time from chest pain to arrival at the ER was 9.5 h. That is far beyond the range recommended internationally. Males, smokers, and younger age patients were significantly more likely to present earlier than their counterparts (*p* < 0.05).

**Conclusions:**

Both educational level and health awareness of cardiac symptoms are associated with early presentation to the ER in STEMI patients. Developing health awareness activities targeting various population groups regarding cardiac symptoms and how to deal with them and including health education in different educational curricula are recommended.

## Introduction

Cardiovascular diseases are the number one cause of death worldwide [1].This includes both acute and chronic heart diseases. The generally used term (heart attack) is commonly used by the public to describe sudden heart problems. Acute coronary syndrome is the most common type of sudden cardiac problems. Acute coronary syndrome is further categorized based on several factors including the clinical picture of the patient, electrocardiogram (ECG), echocardiography, and cardiac enzymes. These categories are managed differently in the emergency room (ER) at the hospital and afterward. The most serious category is ST-segment elevation myocardial infarction (STEMI). This usually indicates total occlusion of a coronary artery. STEMI is a very serious medical condition that might be fatal in many cases [2].

There are two major methods of revascularization in STEMI cases. The old method uses thrombolytic therapy to find a path for the blood through the occluded coronary vessel of the heart. This treatment strategy comes with possible serious side effects and multiple contraindications. The other currently ideal strategy is doing coronary angiography to determine the occluded vessel and open it. Time is the most important factor in the management of STEMI patients. The later the revascularization of the occluded vessel, the more damage the cardiac muscles are exposed to with worse complications. Several studies have been done, and guidelines have been developed to define a protocol for treatment of such serious conditions [3].

The guidelines state that 90 min is the maximum time that the medical teams have to reopen the occluded vessel in the catheterization lab using primary percutaneous intervention (primary PCI). This time starts at the first medical encounter with the patient. All the efforts should be exerted to keep this time frame as short as possible. Several strategies have been adopted in different cardiac centers and health systems worldwide to address this time frame. This includes communication systems between the ambulances and hospitals to bypass the ER and move directly to the catheterization lab. Another modality is STEMI code where specialized teams are always ready and on call for such situations [4].

Despite the previously mentioned strategies to keep this time frame short, there is another period shown to affect the results and complications happening to STEMI patients. It is the time from the onset of symptoms until the time the patient seeks medical help either by calling the ambulance or visiting the ER of a nearby hospital. Some recent studies have addressed this period and attempted to identify the factors that affect it. These factors include the neighborhood the patient is living in and its distance from medical services. It also includes the transportation means, effectiveness of referral systems and emergency ambulance services, severity of the chest pain caused by the myocardial infarction, and patient awareness and education about such medical emergencies [2, 5].

The level of education is widely recognized as a significant factor influencing the outcomes of various medical conditions. Numerous studies have concluded its impact, particularly in emergency medical situations such as acute coronary syndromes, including STEMI. Medical awareness is shaped by general education, including schooling and scientific degrees, knowledge of the available medical services and their functions, personal or familial exposure to similar conditions, or working in the medical field [5, 6]. This study aims to assess the relationship between the educational level, health awareness, and the presentation time to the ER or medical service.

## Methods

### Study design and setting

This was a cohort observational study conducted at the National Heart Institute in Egypt gathering data of the patient at the first medical encounter at the hospital ER, after the intervention time, and before discharge from the hospital with a follow-up during hospital stay.

- *Site*: National Heart Institute (NHI) in Egypt (the highest flow center in primary coronary intervention (PCI) in Egypt with a mean rate of 15 primary PCI per day)
- *Study period*: The study recruited patients over *6 months* from March 2023 to September 2023.

### Definition of terms

*Educational level*: The educational level was categorized as follows:

- *High*: Graduation from university
- *Mid*: High school
- *Low*: Less than high school

*Health awareness* was based on different factors including whether the patient was exposed to a similar situation of myocardial infarction with a close relative or a family member or whether he was exposed to health awareness information on chest pain and myocardial infarction before or having a family history of a similar situation. The patient was considered health-aware if any of the previous factors were positive. This means that he has a basic idea of the risks of chest pain or the symptoms of myocardial infarction.

### Participants

- *Inclusion criteria*
- Acute coronary syndrome (ST-segment elevation myocardial infarction STEMI) patients
- Age 18–75 years
- *Exclusion criteria *(Table 1)

**Table 1:** Exclusion criteria

Type of patient	Reason for exclusion
Transferred patients from other centers or by ambulance	More than 120 min of transfer time is an indication for lytic not primary PCI therapy in the guidelines Time delay cannot be due to patient awareness alone
Older than 75 years	Might need assistance with transportation to the hospital
Shocked or unconscious at the time of presentation	
Type II or complicated diabetes more than 10 years or Type I or on insulin	Diabetes mellitus leads to neuropathy and impaired sensation of pain

### Statistical analysis

The Stata 17 software was used to analyze the data. Data was categorized based on whether they were numerical or categorical data. The mean was used to describe continuous data with normal distribution. In skewed data, the median and interquartile range were used. The independent *t*-test was used to assess the association between health awareness and the time of arrival to the hospital since the maximum intensity of chest pain. The chi-square test was used to test the association between health awareness and the categorical variables. The level of significance was determined to be *p* < 0.05.

## Results

**Fig. 1:**
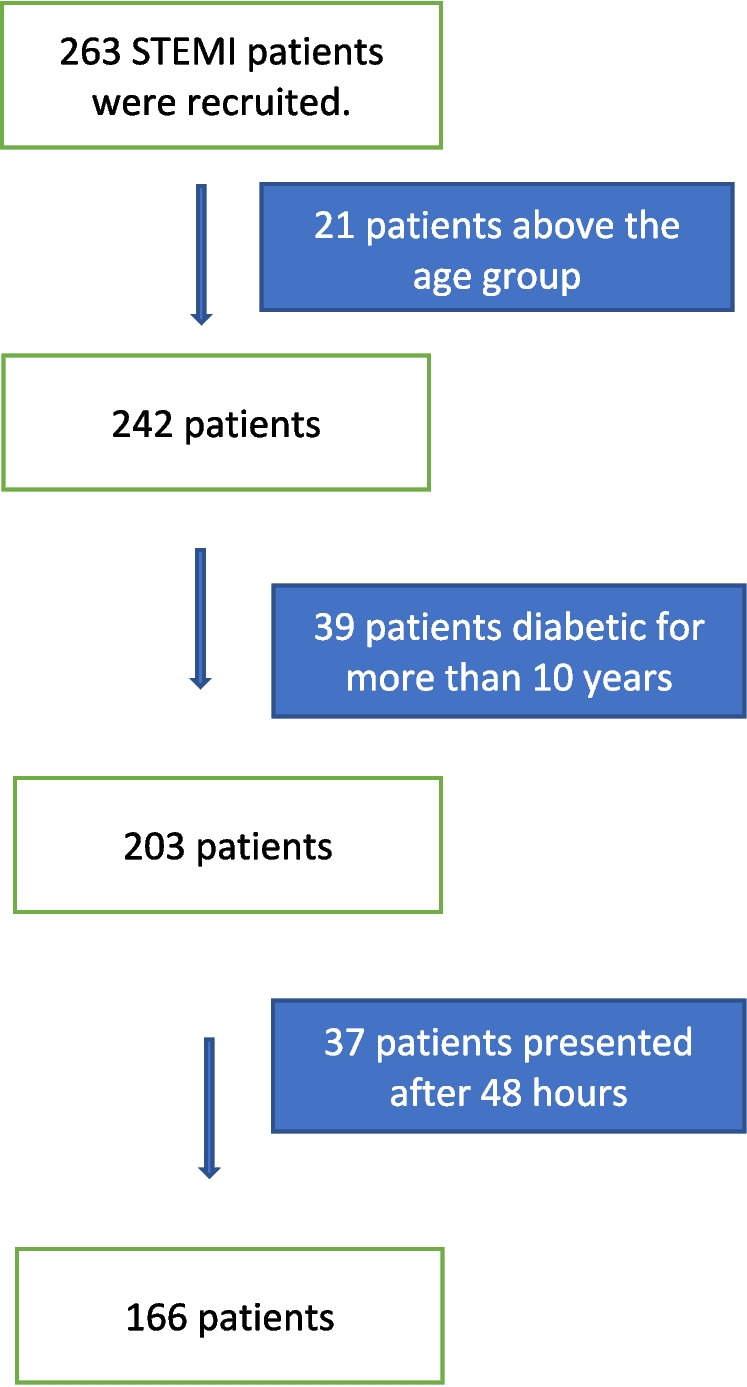
Study sample after applying the exclusion criteria

During the study period, 263 patients who presented to the ER of the National Heart Institute with STEMI were recruited. The sample size was 166 STEMI patients between 18 years of age after applying the exclusion criteria (Fig. 1).

The age range of the group was wide with the youngest person presenting aged 28 years old while the oldest person was 74 years old. The mean age was 56.5 years (median 58 years, skewness − 0.31, kurtosis: 2.48). Most of the study population comprised 132 males (79.5%) and 34 females (20.5%).

Several ischemic heart disease risk factors were reported in the study population. Less than half of the participants were diabetic (46%). A similar percentage was hypertensive (45%), while 60% of the participants were smokers. Almost one-third of the group had dyslipidemia (30.1%). A small percentage (9.6%) of the studied group had a positive family history of ischemic heart disease.

Anterior STEMI was the most commonly diagnosed STEMI type with 98 patients representing almost 59% of the sample. Inferior STEMI was diagnosed in around 38% of the patients. Isolated posterior STEMI was detected only once, and lateral STEMI was diagnosed in five patients (Table 2).

**Table 2:** Risk profile of the patients presenting with STEMI at the National Heart Institute, Cairo, Egypt, 2023 (*n* = 166)#

Variable	Yes		No	
	**(*n*)**	**(%)**	**(*n*)**	**(%)**
Sex (male)	132	79.5	34	20.5
Type of STEMI (anterior)	98	59.0	68	41.0
Diabetes mellitus	76	45.8	90	54.2
Hypertension	74	44.6	92	55.4
Smoker	99	59.6	67	40.4
Dyslipidemia	50	30.1	116	69.9
Positive family history of ischemic heart disease	15	9.6	151	90.4

^#^This number includes the study population after the application of the inclusion and exclusion criteria to patients presenting with STEMI at the National Heart Institute, Cairo, Egypt, 2023

**Fig. 2:**
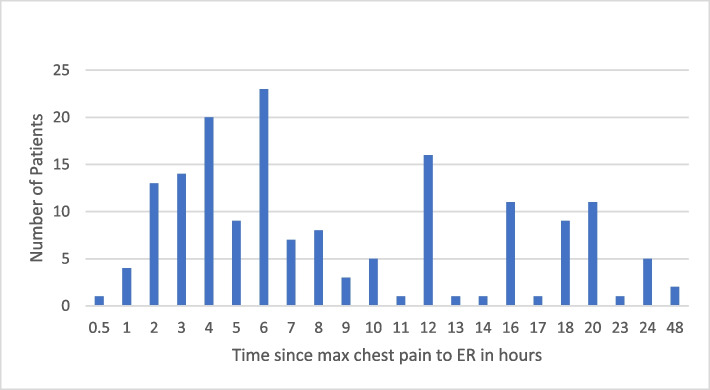
Distribution of time from the maximum intensity of chest pain until reaching the emergency room

The mean arrival time to the emergency room (ER) was 9.5 h, ranging from 8.2 to 10.6 h. The median was 6 h (skewness: 1.9, kurtosis: 9.1). The minimum time was 1 h for a single patient, while many patients presented much later. This is illustrated in Fig. 2. This study recruited only patients with a maximum time to ER since chest pain of 48 h because they were the ones admitted to the catheterization laboratory and had coronary revascularization. The time spent to get the patient to the cath laboratory was variable with a mean of 2.5 h (95% *CI*: 2–2.9 h).

Most of the study population resided near the hospital, and time was calculated from the time since maximum intensity of chest pain until arrival at the ER. Most of the study population were close to the NHI when experiencing chest pain. The mean drive travel time by car was almost 40 min (95% *CI*: 37–42 min).

**Fig. 3:**
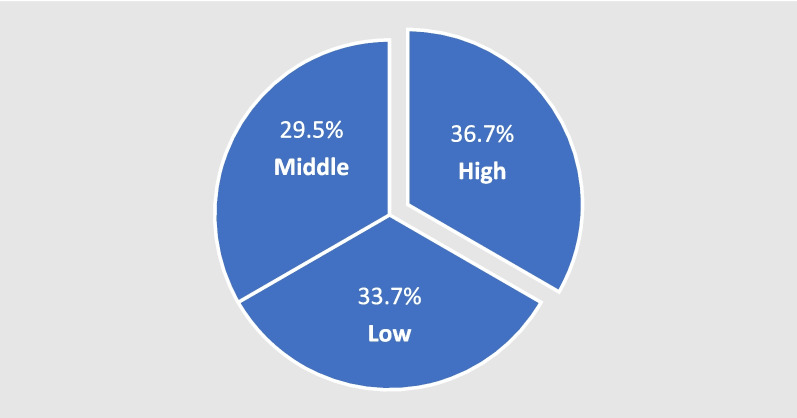
Educational level in the study group (*n* = 166)

The distribution of educational level was balanced in the study sample with the highest number being highly educated people (*n* = 61, 36.8%) holding university degrees or higher. There were 56 patients (33.7%) with a low educational level and 49 patients (29.5%) with an intermediate educational level, with a diploma or high school degree (Fig. 3).

From the total sample, 51 patients with various educational levels were aware of heart attack symptoms. This was based on their history of IHD, exposure to a similar situation with a relative or close friend, exposure to a health awareness campaign or material, or having a family history of a similar situation. Only 31% of the admitted patients received any health awareness information regarding the nature and risks of chest pain and how to react if this happens There was no statistically significant difference between the health-aware and non-health-aware patients in their risk profile (Table 3).

**Table 3:** Health awareness and patients’ risk profile of patients with STEMI presenting at the National Heart Institute (NHI), Cairo, Egypt, 2023 (*n* = 166)

Variable	Aware *n* (%)	Non-aware *n* (%)	*p*-value
Age below 55 (**years**)	51 (30.7)	115 (69.3)	0.7
Sex			
Males (***n* = 132**)	41 (31.1)	91 (68.9)	0.85
Female (***n* = 34**)	10 (29.4)	24 (70.6)	
Diabetes mellitus (***n* = 76**)	24 (31.6)	52 (68.4)	0.8
Smoker (***n* = 99**)	29 (29.3)	70 (70.7)	0.6
Dyslipidemia (***n* = 50**)	19 (38.0)	31 (62.0)	0.1
Hypertension (***n* = 74**)	23 (31.1)	51 (68.9)	0.9
Anterior ST-segment elevation myocardial infarction (***n* = 98**)	25 (25.5)	73 (74.5)	0.08
Educational level (***n* = 166**)	51 (30.7)	115 (69.3)	0.43

The chi-square test was used in categorical data

^*^The *p*-value was considered significant if < 0.05. The independent *t*-test was used in continuous data

In terms of the time of presenting to the ER, there is a statistically significant difference between the early- and late-presenting groups in their age, sex, and smoking history. The mean age in the early-presenting group was 55.2 years (*SD* = 9.5) and 59 years (*SD* = 9.8) in the late-presenting group. The difference is only 3.8 years and is not clinically significant even if statistically significant (Table 4). The sex difference in early and late presentation was clear in the male group with 93 males presenting early and 39 presenting late. This was not the situation among female patients with 16 females presenting early and 18 presenting late (*p* = 0.01). Finally, it was detected that 71 smokers presented early while 28 presented late (*p* = 0.046).

**Table 4:** The relationship between time of presentation (early versus late presenters) and risk profile (*n* = 166)

Variable	Early *n* (%)	Late *n* (%)	*p*-value
Age (**years**)	55.2 (*SD* = 9.5)	59 (*SD* = 9.8)	0.01*
Sex			
Males (***n***)	93 (70.5)	39 (23.5)	0.01*
Female (***n***)	16 (47.1)	18 (52.9)	
Diabetes mellitus (***n***)	47 (61.8)	29 (38.2)	0.3
Hypertension (***n***)	46 (85.2)	8 (14.8)	0.3
Dyslipidemia (***n***)	30 (60.0)	20 (40.0)	0.3
Smoker (***n***)	71 (71.7)	28 (28.3)	0.046*
Anterior ST-segment elevation myocardial infarction (***n***)	62 (63.3)	36 (36.7)	0.4
Residence (nearby hospital)	101 (60.8)	65 (39.2)	0.08

^*^The *p*-value was considered significant if < 0.05. The independent *t*-test was used in continuous data. Chi-square test was used in categorical data

^*^A cutoff point of 12 h was set to differentiate between early and late presenters

The distance of the patient from the hospital when experiencing chest pain was nonsignificantly associated with an earlier presentation to the ER. Patients residing near the hospital did not show up earlier to the emergency room (*p* = 0.08). Most of the study population (STEMI patients receiving primary PCI) were from nearby areas to the hospital represented in the Greater Cairo zone.

Health awareness was highly significantly associated with early presentation to the emergency room in patients with STEMI (*p* < 0.001) (Table 5).

**Table 5:** The relationship between health awareness of patients with STEMI and presentation time (*n* = 166)

Variable	Aware *n* (%)	Non-aware *n* (%)	*p*-value
Early presentation	44 (26.5)	64 (38.5)	0.000*
Late presentation	7 (4.2)	50 (30.1)	

^*^The *p*-value was considered significant if < 0.05. These results were obtained using the chi-square test

^*^A cutoff point of 12 h was set to differentiate between early and late presenters

The educational level with its different categories was highly and significantly associated with the early presentation to the emergency room in patients with STEMI (*p* < 0.001) (Table 6).

**Table 6:** The relationship between the educational level of patients with STEMI and presentation time to the emergency room (*n* = 166)

Presentation time (early/late)	Educational level			Total (*n*)	*p*-value
	**High (n)**	**Low (n)**	**Middle (n)**		
**Early**	54	27	28	109	*p* < 0.001*
**Late**	7	29	21	57	*p* < 0.001*
**Total**	61	56	49	166	

^*^The difference was considered significant if the *p*-value was < 0.05. These results were obtained using the chi-square test which is highly statistically significant (*p* < 0.001)

## Discussion

This study examined the relationship between health awareness levels and clinical situations. It specifically assesses the relationship between awareness and time to ER in STEMI patients. It is part of our larger cohort study on the relationship between education/health awareness and outcomes of myocardial infarction patients. Patients’ profiles are consistently studied in all international and national studies discussing health awareness and cardiovascular diseases, especially heart attacks, to determine mitigatable risk factors. While heart attacks commonly affect older adults, they are not limited to this age group. The mean age in our study was in the 50 s [55–59] in both the aware and non-aware groups respectively. This population segment suffers from some diseases such as hypertension, diabetes mellitus, positive family history, and dyslipidemia. Additionally, many of them engage in risky behaviors such as smoking. The studied age group usually ranges from 18 to 65 years of age. There is evidence suggesting a relationship between health awareness of cardiovascular diseases (CVDs) and age, with some studies detecting significantly higher levels of awareness among older age groups [7, 8]. Both referenced studies specifically examined awareness related to hypertension (HTN). An interesting study in the USA discussed the role of age, sex, and race in health awareness with a particular focus on females. Only 38% declared that their physicians had mentioned anything about heart disease to them, and women below 45 years had lower health awareness levels [9].

Health awareness of a person is known to be influenced by several factors, such as the person’s educational level, his financial status and monthly income, housing and living facilities, and other factors. The education of a person guides his behavior in various situations. When it comes to health-related conditions, especially a critical one like a heart attack (myocardial infarction), the person needs to use all his intellectual capacities to act promptly and correctly. The exposure to similar situations with a close friend or a family member experiencing MI makes the patient more aware of his symptoms and how to act. The exposure to health awareness materials related to chronic noncommunicable diseases makes the patient aware of the risk he might be in. These materials might include outreach campaigns, health videos by doctors on social media platforms, or directly through word of mouth from the family or personal physician.

Amin et al. (2014) [10] developed a questionnaire to assess the factors contributing to public health awareness regarding CVD. This questionnaire was additionally validated by another study [11]. In our study, we used all the factors mentioned in this questionnaire. These include previous history of chronic illness, cardiovascular disease (CVD), family history of CVD, age, educational level, and exposure to awareness campaigns. They detected that either suffering from or having a family history of CVDs or diabetes mellitus (DM) predicts a higher level of health awareness.

The educational level correlation with health awareness was the core of several international studies. Most of these studies detected a significant correlation between the educational level and health awareness as related to cardiovascular diseases including DM, HTN, and stroke. Different studies had different categorizations for the educational levels, however. For example, a study in southwest China [12] classified it into different nine classes from never being to school up to receiving postgraduate studies. In Poland, Sękowski et al. [13] divided it into primary school level, vocational school, secondary school, or higher degree of education. That was close to what has been applied in our study. A similar categorization was used by Ahmed et al. [11] in their study on the impact of health awareness on heart attack and cerebrovascular stroke. They categorized it into lower school, secondary school, undergraduate degree, and postgraduate studies. Our study detected a positive significant correlation between the level of formal education and time of presentation to the ER in myocardial infarction cases despite detecting no correlation between health awareness and level of education.

Health awareness is not only important in myocardial infarction or cardiac diseases. It is very important in stroke patients where the time window to revascularize is even narrower. Further, studies confirmed the role of educational level and awareness in chronic diseases such as diabetes [14–16] and hypertension [7, 8] to avoid developing complications.

Early presentation to the ER or catheterization lab is crucial in managing myocardial infarctions. Evidence has consistently shown that the shorter the total ischemic time from the onset of chest pain until revascularization, the better the outcomes of the myocardial infarction [3, 4]. Many patients experience chest pain for a while before they start seeking help. Many become confused it might be gastritis, myositis, flu, or common cold. They might seek help from nonspecialized physicians or pharmacists or take over-the-counter painkillers. Unfortunately, all these practices do not treat the ongoing pathological process of coronary occlusion and myocardial deprivation of blood and only delay the appropriate treatment.

Our focus was on the time from pain to ER. Several factors play a role in this including awareness of the problem itself that it might be cardiac and require intervention. The mean time from chest pain to arrival at the ER in this study was 9.5 h. That is far beyond the international recommendation. Early-presenting patients especially those within the first 4–6 h of chest pain are expected to get the most benefit of intervention and revascularization because myocardial damage is the least. A recent systematic review in 2023 by Kennedy et al. [17] including over 7000 patients concluded that patients above 60 years presenting after 4–6 h of total ischemic time develop coronary no-reflow at higher rates than patients presenting earlier, which increases the likelihood of PCI failure. Bessenov et al. (2021) [18] detected similar results with patients presenting after 6 h experiencing a higher incidence of no-reflow, while those presenting beyond 3 h experienced a higher incidence of complications and mortality. The factors leading to longer ischemic times in their study were older age, female gender, and chronic kidney disease.

Potential delay reasons in our study were attributed to the lack of health awareness in the Egyptian population. Only 31% of the admitted patients received any health awareness information regarding the nature and risks of chest pain and how to react if this happens. Additionally, the lack of trust in the governmental hospitals and concerns over the quality of service by some patients may have played a role in delaying their call for help particularly if the pain is not severe. Research on reasons for prehospital delay showed that for older patients requiring assistance to reach the hospital, the lack of caregivers caused further delays [19]. Distance and transport to the nearest hospital providing the primary PCI service 24 h a day are another important factor in determining the time to the ER [20]. The severity of pain and the patient’s perception of its nature and seriousness played major roles in deciding whether and when to seek help [21, 22].

A remarkable finding in our study was that smokers presented to the ER earlier than nonsmokers. One possible explanation is that smokers have been specifically targeted by community health awareness efforts, with “smoking hazard warnings” prominently displayed on cigarette packs. Another explanation may be that smokers recognize that they are engaging in risky behavior that increases their risk of heart attack [23], making them more likely to seek medical advice at a lower threshold in cases of chest pain or other heart attack symptoms.

Additionally, the gender difference in the time of presentation to the ER was statistically significant. Females presented later than males despite having the same risk profile and STEMI diagnosis. This delay could be attributed to cultural factors, particularly in conservative regions such as the Imbaba district where the NHI is located, where females depend on male caregivers to accompany them to the hospital, especially at night. Additional time may be spent searching for safe suitable transportation. Furthermore, global data suggests that women are usually underdiagnosed with ischemic heart disease [24]. As mentioned earlier, only 38% of the females declared that their physicians had mentioned anything about heart disease to them.

Finally, data discussing the correlation between health awareness or health literacy in acute coronary syndrome with a particular focus on time to ER is limited. A systematic review of 86 studies detected supportive data to our study stating that lack of proper health knowledge of cardiac symptoms led to delayed presentation to ER specially in women and older groups. This led to a higher incidence of complications including death [25]. A cross-sectional study from Munich assessed 486 patients presenting with acute myocardial infarction confirming that higher knowledge of myocardial infarction shortened the median delay time in both men and women [26]. On the contrary, a cross-sectional study evaluating 200 patients from Saudi Arabia concludes a significant correlation between prehospital delay and previous information on acute coronary syndrome [27].

Educational level was one of the major factors determining pain to ER time. A study from Iran on acute myocardial infarction patients detected significant delay in presentation to ER among uneducated STEMI patients [28]. A multicenter study from Norway detected that low educational levels in both the patients and their partners prolonged time to ER [29]. Even during the COVID-19 pandemic, educational level played a major role in the time patients consumed to seek medical care in cases of acute coronary syndrome [30]. These results support the data found in our study confirming that both health awareness and educational level separately play major roles in shortening prehospital time in patients with acute myocardial infarction.

### Study limitations

The study was a single-center study with a relatively small sample size. The study did not assess patients presented by ambulance or transferred patients from other centers, so a portion of STEMI patients were missing. Additionally, the acute nature of the disease did not allow for a full interview of the patient regarding his knowledge of cardiac diseases.

## Conclusions

Education level and health awareness of cardiac symptoms are associated with early presentation to the emergency room (ER) in STEMI patients. Males, younger patients, and smokers were significantly more likely to present earlier to the ER. It is highly recommended to develop health awareness activities targeting different groups of the population regarding cardiac symptoms and how to deal with them. Additionally, it is highly recommended to include health awareness in the curricula at different levels of education and to address gender inequality issues in the accessibility of Egyptian healthcare services, especially in emergencies.

## Data Availability

The data that support the findings of this study are available from the corresponding author upon request.
